# Intestinal microbiome changes and mechanisms of maintenance hemodialysis patients with constipation

**DOI:** 10.3389/fcimb.2024.1495364

**Published:** 2024-11-11

**Authors:** Aiping Zhang, Shilei Chen, Yanqin Zhu, Mengqi Wu, Bin Lu, Xin Zhou, Yan Zhu, Xinyu Xu, Hong Liu, Fenggui Zhu, Riyang Lin

**Affiliations:** ^1^ Department of nephrology, Hangzhou Traditional Chinese Medicine Hospital of Zhejiang Chinese Medical University, Hangzhou, Zhejiang, China; ^2^ Department of General Medicine, Hangzhou Xihu District Zhuantang Street Community Health Service Centre, Hangzhou, Zhejiang, China; ^3^ Department of Oncology, Hangzhou Traditional Chinese Medicine Hospital of Zhejiang Chinese Medical University, Hangzhou, Zhejiang, China; ^4^ Key Laboratory of Kidney Disease Prevention and Control Technology, Hangzhou, Zhejiang, China

**Keywords:** Constipation, gut microbiome, 16S rRNA, Maintenance haemodialysis (MHD), Intestinal biomarker

## Abstract

**Background:**

Constipation is a common symptom in maintenance hemodialysis patients and greatly affects the quality of survival of hemodialysis patients. Fecal microbiota transplantation and probiotics are feasible treatments for functional constipation, but there is still a gap in the research on the characteristics of gut flora in patients with maintenance hemodialysis combined with constipation. The aim of this study is to clarify the characteristics of the intestinal flora and its changes in maintenance hemodialysis patients with constipation.

**Methods:**

Fecal samples were collected from 45 participants, containing 15 in the maintenance hemodialysis constipation group,15 in the maintenance hemodialysis non-constipation group and 15 in the healthy control group. These samples were analyzed using 16S rRNA gene sequencing. The feature of the intestinal microbiome of maintenance hemodialysis constipation group and the microbiome differences among the three groups were elucidated by species annotation analysis, α-diversity analysis, β-diversity analysis, species difference analysis, and predictive functional analysis.

**Results:**

The alpha diversity analysis indicated that maintenance hemodialysis constipation group was less diverse and homogeneous than maintenance hemodialysis non-constipation group and healthy control group. At the genus level, the top ten dominant genera in maintenance hemodialysis constipation group patients were Enterococcus, Escherichia-Shigella, Bacteroides, Streptococcus, Bifidobacterium, Ruminococcus_gnavus_group, Lachnospiraceae_unclassified, Faecalibacterium, Akkermansia and UCG-002. Compared with non-constipation group, the Enterococcus, Rhizobiales_unclassified, Filomicrobium, Eggerthella, Allobaculum, Prevotella_7, Gordonibacter, Mitochondria_unclassified, Lachnoanaerobaculum were significantly higher in constipation group (p<0.05). Compared with non-constipation group, the Kineothrix, Rhodopirellula, Weissella were significantly lower in constipation group (p<0.05). The predictive functional analysis revealed that compared with non-constipation group, constipation group was significantly enriched in pathways associated with pyruate metabolism, flavonoid biosynthesis.

**Conclusions:**

This study describes for the first time the intestinal microbiome characteristics of maintenance hemodialysis patients with constipation. The results of this study suggest that there is a difference in the intestinal flora between maintenance hemodialysis patients with constipation and maintenance hemodialysis patients without constipation.

## 1 Introduction

Renal failure is defined as a glomerular filtration rate <15 ml/min/1.73 m2, which can be treated with renal replacement therapy (meaning dialysis or transplantation) or supportive care (Stevens, 2013). Maintenance hemodialysis (MHD) is one of the main treatments used for end-stage renal disease (ESRD), with approximately 89% of dialysis patients receiving hemodialysis (HD) worldwide (Pecoits-Filho et al., 2020). As of 31 December 2017, 62.7% of the 746,557 prevalent cases of ESRD in the US were HD patients. In Europe and worldwide, there are approximately 350,000 and 3 million HD patients, respectively, and numbers are anticipated to over 5.4 million globally by 2030, not including the large number of patients who are unable to access HD treatment for economic reasons (Basile et al., 2021).

A previous report showed patients on MHD are more likely to suffer from varying degrees of gastrointestinal symptoms, such as constipation, nausea and vomiting, loss of appetite, and bloating, etc., of which constipation is one of the very frequent complications, with an incidence of 53% (Murtagh et al., 2007). The occurrence of constipation is mainly attributed to dietary restrictions, lack of exercise, use of calcium-phosphorus binding agents and potassium-lowering resins, accumulation of toxins in the body, the suppression of the urge to defecate during HD, and dysbiosis of intestinal flora (Yasuda et al., 2002). Constipation has a number of adverse effects on MHD patients, including inadequate nutritional intake, negative emotions such as sadness and anxiety, increased risk of hypotension during dialysis, cardiovascular accidents, and even increased accumulation of toxins such as creatinine and urea nitrogen in ESRD patients (Zhang JJ and Shen, 2018). Clinical solutions to the problem of constipation in maintenance hemodialysis patients have gained attention. Osmotic and excitatory laxatives are considered first-line drugs for the relief of constipation in adults (Krogh et al., 2017). Osmotic laxatives are widely used in chronic kidney disease (CKD), but their role may be limited, especially in HD patients. Moreover, osmotic laxatives containing magnesium and sodium may induce adverse renal and metabolic disorders (Heher et al., 2008). Therefore, contact laxatives are commonly used in HD patients suffering from constipation. However, diarrhea, dehydration and electrolyte disorders are common side effects of contact laxative use (Oster et al., 1980). One study found a significant dose- and duration-dependent relationship between contact laxative use and increased risk of arteriovenous fistula maturation failure (Hoang Anh et al., 2022). Additionally, long-term laxative use can increase the risk of adverse cardiovascular events and death (Kubota et al., 2016; Hoppe et al., 2019; Honda et al., 2021).

Research has indicated that intestinal flora dysbiosis is an important cause of the occurrence and development of constipation, and conversely, constipation will make the patient’s gut microbiota dysbiosis, which is a mutually reinforcing process (Huang, 2020; Lydia et al., 2022). Individuals with CKD or ESRD have a unique microbial community in the gut that includes an overgrowth of duodenal and jejunal bacteria, an overgrowth of certain aerobic bacteria, and altered genera of commensal bacteria, leading to a dysfunctional gut ecosystem (Vaziri et al., 2013; Ramezani et al., 2016), and the development of chronic gastrointestinal symptoms such as constipation and loss of appetite. There have been a number of studies on the use of synbiotics to reduce uremic toxins and relieve constipation. However, there is a wide variation in the effect obtained using different types of synbiotics, suggesting the value of specifying the type, dose and duration of synbiotics (Nakabayashi et al., 2011; Rossi et al., 2016; Cosola et al., 2021; McFarlane et al., 2021; Nguyen et al., 2021). Therefore, the fecal microbiota analysis is need to be performed to determine the most suitable synbiotics for MHD patients. In addition, numerous clinical Trials have demonstrated that fecal microbiota transplantation (FMT) reduces functional constipation. However, to the best of our knowledge, no study has reported the characteristics of gut flora in maintenance hemodialysis patients with constipation. Therefore, this research gap is urgent to be addressed.

The aim of this study was to examine the differences in the distribution and abundance of intestinal microbiome among the three groups, including maintenance hemodialysis patients with constipation, maintenance hemodialysis patients without constipation and healthy individuals. And to identify the dominant intestinal flora in MHD patients with constipation, so as to explore possible mechanisms of MHD patients with constipation.

## 2 Methods

### 2.1 Study design

In this study, a prospective clinical cohort study was conducted to analyze the bacterial flora in the stools of maintenance hemodialysis patients with constipation, and hemodialysis patients who were not constipated and normal healthy individuals were used as controls. This study starts in January 2023 and lasts for 1 year. All samples in this study were obtained from the feces of participants in Hangzhou, China.

### 2.2 Study participants

Based on the inclusion and exclusion criteria, 336 patients undergoing maintenance hemodialysis at the Hemodialysis Centre of Hangzhou Hospital of Traditional Chinese Medicine (TCM) were screened. A questionnaire survey of constipation-related symptoms was conducted on these 336 patients. Then, 100 MHD patients who met the Rome-IV diagnostic criteria for constipation were screened out (**Supplementary Material 1**). From these 100 patients, 15 was randomly selected as the maintenance hemodialysis constipation group (MHDCG). And the same number of people were set up in the maintenance hemodialysis non-constipated group (MHDNCG) and the healthy control group (HCG) respectively. The HCG came from Hangzhou Hospital of Traditional Chinese Medicine and were older than 18 years old, and the results showed that they were in good health, had no gastrointestinal symptoms, and had not taken any medications in the past month. The study was ethically reviewed by the Research Ethics Committee of Hangzhou Hospital of TCM.

The inclusion criteria were as follows:

1. Maintenance hemodialysis treatment for more than 3 months with stable condition;
2. Patients older than 18 years of age;
3. Maintenance hemodialysis patients who were conscious, had no language communication barriers; gave informed consent and signed the Patient Informed Consent Form.

The exclusion criteria were as follows:

1. Those who have had infections or stress conditions such as abdominal pain and diarrhea, coughing and sputum in the last month, and who have used antibiotics;
2. History of taking probiotics, prebiotics, antibiotics or proton pump inhibitors within the last 1 month;
3. Presence of organic gastrointestinal disorders (e.g. irritable bowel syndrome, intestinal obstruction, intestinal adhesions, etc.), or history of gastrointestinal disorders (e.g. tumors, ulcerative colitis, etc.), or history of gastrointestinal surgery.
4. Patients with incomplete clinical information or who did not provide a fecal specimen on request.

### 2.3 Fecal sample collection and DNA extraction

Fresh fecal specimens were collected from the selected individuals. The fecal specimens were excreted into the aseptic stool tubes and moved to a −80°C refrigerator for storage within 2 h after sampling. The total DNA of microbiome was extracted by cetyltrimethylammonium bromide (CTAB) method, and the purity and concentration of DNA were detected by agarose gel electrophoresis.

### 2.4 16S rRNA gene targeted amplification and sequencing

The use of different primers for polymerase chain reaction (PCR) amplification was selected based on the different project, the PCR products were purified from AMPure XT beads (Beckman Coulter Genomics, Danvers, MA, USA) and quantified by Qubit (Invitrogen, USA). PCR amplification products were detected by 2% agarose gel electrophoresis. The purified PCR products were evaluated using Agilent 2100 Bioanalyzer (Agilent, USA) and Illumina (Kapa Biosciences, Woburn, MA, USA) library quantification kits, and the acceptable library concentration should be above 2nM.The qualified sequencing libraries (Index sequences are not reproducible) were diluted in a gradient, mixed according to the required sequencing volume in the appropriate ratio, and denatured by NaOH to single-stranded for sequencing;2×250bp double-end sequencing was carried out using the NovaSeq 6000 Sequencer, and the corresponding reagent was NovaSeq 6000 SP Reagent Kit (500 cycles).

### 2.5 Statistical analysis

Based on the Amplicon Sequence Variant (ASV) sequence files, the SILVA database was used to annotate the species with the NT-16S database, and the abundance of each species in each sample was counted according to the ASV abundance table. Based on the obtained ASV feature sequences and ASV abundance tables, alpha and beta diversity analyses were performed. The alpha diversity analysis was based on the observed_species, shannon, simpson, chao1, pielou_e indices to assess the intra- and inter-group diversity. Beta diversity was assessed by calculating the weighted_unifrac distance, and PCA and PCoA analyses were used to assess the diversity between groups. Linear discriminant analysis Effect Size (LEfSe) analysis was used to find biomarkers. Different statistical methods were selected for species difference analyses according to the samples: Fisher’s exact test was used for samples without biological replicates comparison; Mann-Whitney Utest, for comparison of differences between two groups of samples with biological replicates; Kruskal-Wallis test, comparison between multiple groups of samples with biological replicates. Phylogenetic Investigation of Communities by Reconstruction of Unobserved States (PICRUSt2) v2.2.0b was used to predict potential metagenome functionality based on 16S ASV content for functional analysis. The ASV table and corresponding representative sequences were aligned (NSTI cut-off value of 2) to a reference phylogenetic tree, and the software predicted functional gene families and copy numbers for each specific ASV. The resulting output generated an abundance profile of pathways based on the KEGG database. Differential pathways between groups were identified and presented using STAMP software with t-test. Benjamini-Hochberg FDR-adjusted p values <0.05 were considered significant.

## 3 Results

### 3.1 General characteristics of all participants

In the comparison of general clinical data, MHDCG and MHDNCG were not statistically different in terms of age, gender, ultrafiltration quality and medications taken (p>0.05). MHDCG and HCG were statistically different in age (p=0.003) and not statistically different in gender (p>0.05). There was no statistically significant difference in age and gender for MHDNCG and HCG (p>0.05). In the comparison of test result, MHDCG and MHDNCG did not show statistical difference in blood urea nitrogen, blood creatinine, blood calcium, blood phosphorus and blood uric acid (p>0.05). No statistically significant difference in blood calcium was observed for MHDCG as well as MHDNCG compared to HCG (p>0.05). MHDCG and MHDNCG were statistically different in blood phosphorus compared to HCG (p=0.007, p=0.000) (**Table 1**).

**Table 1:** General characteristics of participants.

	MHDCG(N=15)	MHDNCG(N=15)	HCG(N=15)	t	p
Age(year)	69.20 ± 8.64	62.13 ± 11.39	59.33 ± 8.08	t^a^=1.915; t^b^ =3.231; t^c^ =0.777	p^a^=0.066; p^b^=0.447; p^c^=0.444
Sex (male/female)	10/5	9/6	8/7	/	p^a^=0.500; p^b^=0.710; p^c^=1.000
Blood phosphorus(mmol/L)	1.57 ± 0.35	1.72 ± 0.37	1.26 ± 0.21	t^a^=-1.131; t^b^= 2.972; t^c^= 4.223	p^a^=0.267; p^b^=0.007; p^c^=0.000
Blood calcium (mmol/L)	2.26 ± 0.12	2.26 ± 0.17	2.35 ± 0.18	t^a^=-0.025; t^b^=-1.668; t^c^=-1.422	p^a^=0.981; p^b^=0.106; p^c^=0.166
Blood creatinine(μmol/L)	787.80 ± 204.05	845.53 ± 211.74	/	t^a^=-0.760	p^a^=0.453
Blood uric acid (μmol/L)	441.07 ± 123.71	436.13 ± 86.33	/	t^a^=0.127	p^a^=0.90
Blood urea nitrogen (μmol/L)	22.61 ± 6.67	20.53 ± 8.03	/	t^a^=0.771	p^a^=0.447
Ultrafiltration quality(Kg)	1.89 ± 0.72	1.99 ± 0.93	/	t^a^=-0.351	p^a^=0.729
Taking medication that affects bowel movements (Yes/No)	2/13	5/10	/	/	p^a^=0.195

a: MHDCG vs MHDNCG; b: MHDCG vs HCG; c: MHDNCG vs HCG.

### 3.2 Species annotation analysis

**Figure 1:**
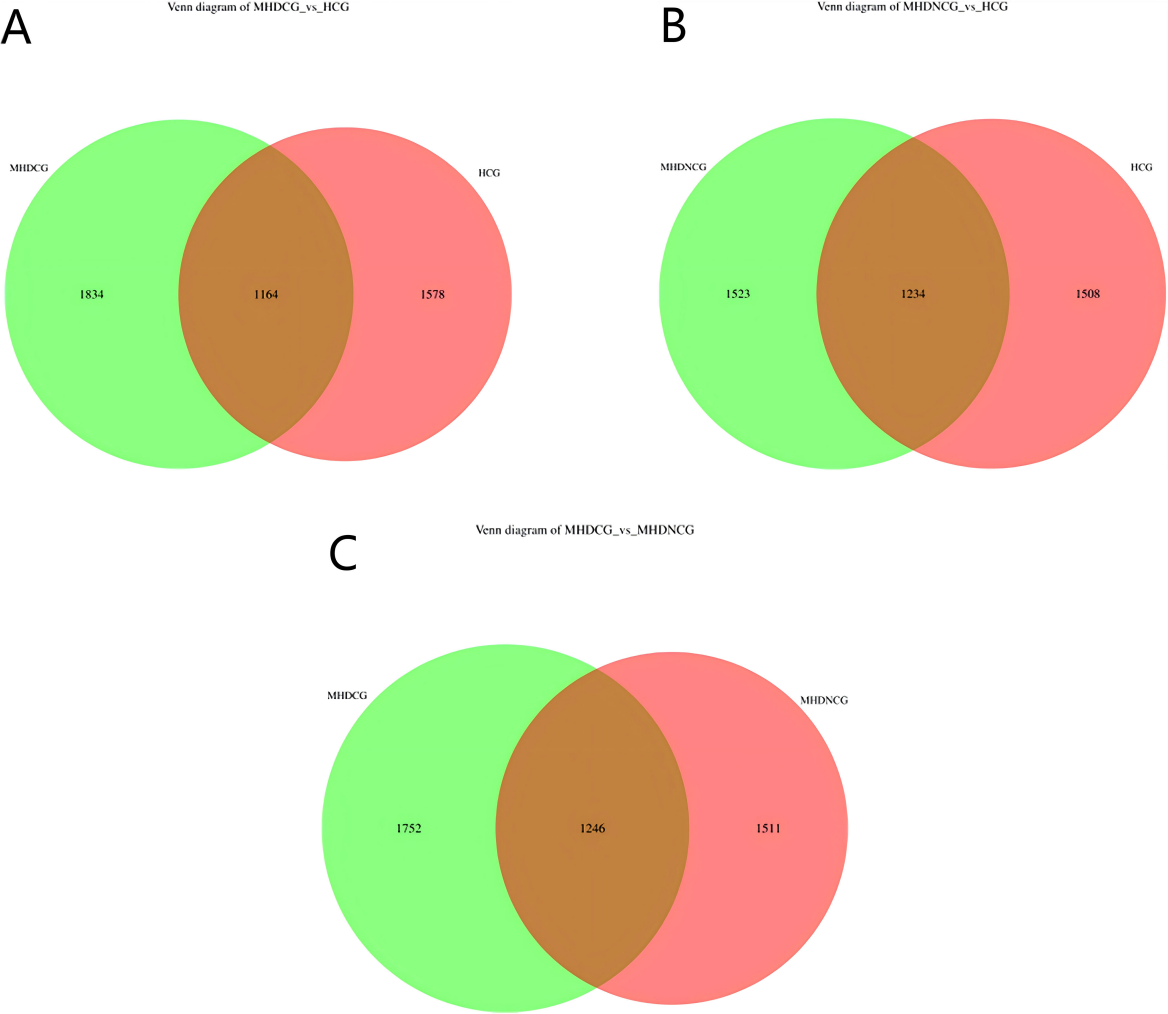
OTU Venn diagram. **(A)** There were 1164 OTUs between the MHDCG and HCG groups, 1834 OTUs specific to the MHDCG group, and 1578 OTUs specific to the HCG group. **(B)** There were 1234 OTUs between the MHDNCG and HCG groups, 1523 OTUs specific to the MHDNCG group, and 1508 OTUs specific to the HCG group. **(C)** There were 1246 OTUs between the MHDCG and MHDNCG groups, 1752 OTUs specific to the MHDCG group, and 1511 OTUs specific to the MHDNCG group.

Using the operational taxonomic unit (OTU) Venn diagram, this study found that there were 1246 OTUs between the MHDCG and MHDNCG groups, 1752 OTUs specific to the MHDCG group, and 1511 OTUs specific to the MHDNCG group. There were 1164 OTUs between the MHDCG and HCG groups, 1834 OTUs specific to the MHDCG group, and 1578 OTUs specific to the HCG group. There were 1234 OTUs between the MHDNCG and HCG groups, 1523 OTUs specific to the MHDNCG group, and 1508 OTUs specific to the HCG group. This tentatively suggests that there are differences in the distribution of intestinal microbiome between MHDCG and MHDNCG, and that the difference in intestinal microbiome species between MHDCG and HCG is greater than that between MHDNCG and HCG (**Figure 1**).

### 3.3 Species diversity analysis

#### 3.3.1 α-diversity analysis

**Figure 2:**
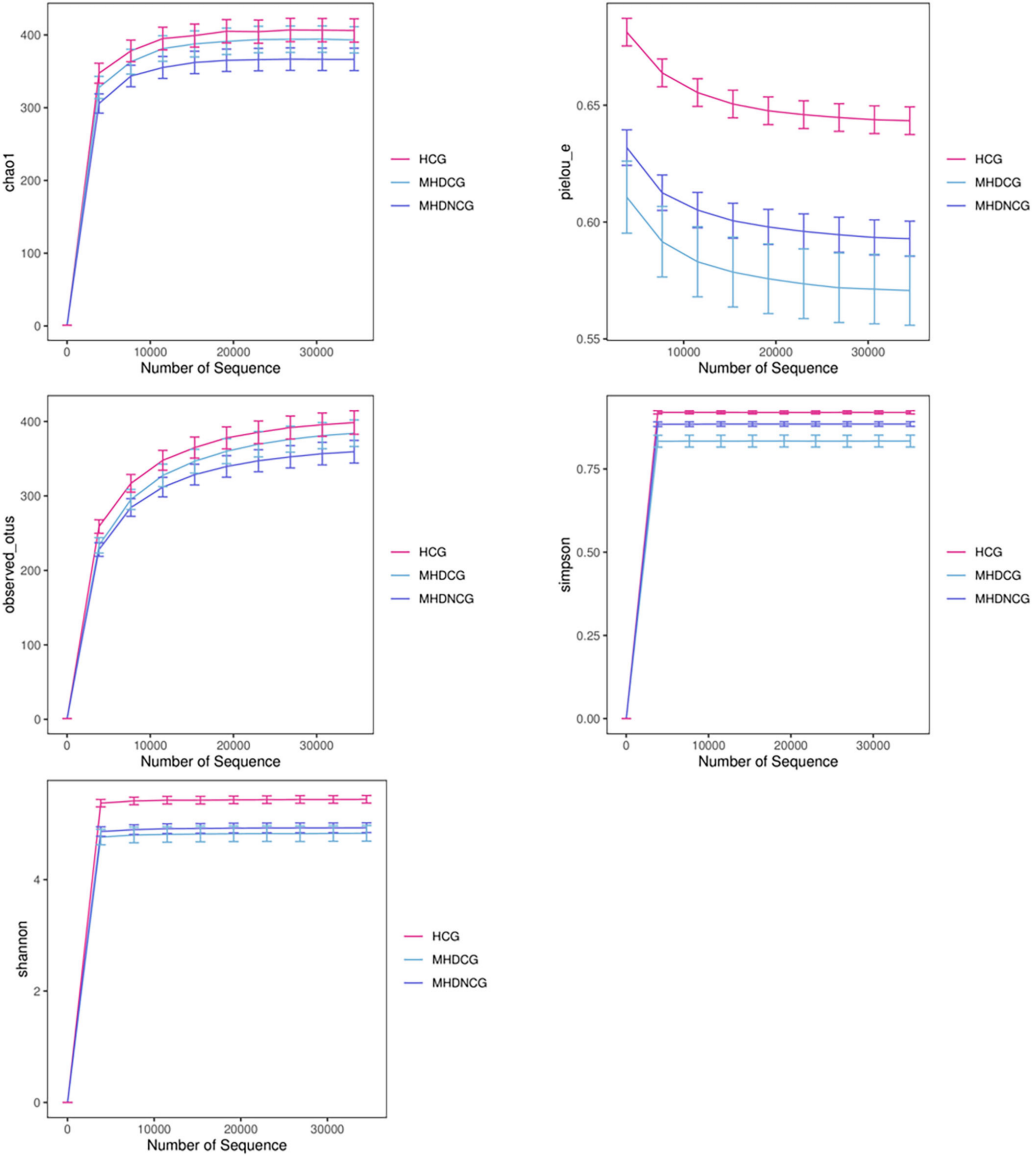
α-diversity analysis. The figures of Chao1 index and observed_species showed that the gut microbial community of MHDCG and MHDNCG contained fewer species than that of the HCG group. The figures of Shannon index and Simpson index showed that the diversity of gut microbial organisms in MHDCG was lower than that of MHDNCG and HCG. The figures of Shannon index and pielou-e index curve showed that the uniformity of gut microbial organisms in MHDCG was lower than that of MHDNCG and HCG.

According to the dilution curve of alpha diversity analysis, combined with Chao1 index and observed_species, this study found that the gut microbial community of MHDCG and MHDNCG contained fewer species than that of the HCG group. Combined with the Shannon index and Simpson index, we found the diversity of gut microbial organisms in MHDCG was lower than that of MHDNCG and HCG. Combined with the Shannon index and pielou-e index curve, we found the uniformity of gut microbial organisms in MHDCG was lower than that of MHDNCG and HCG. In summary, our study suggests that MHDCG have lower diversity and homogeneity than MHDNCG and HCG (**Figure 2**).

#### 3.3.2 β-diversity analysis

**Figure 3:**
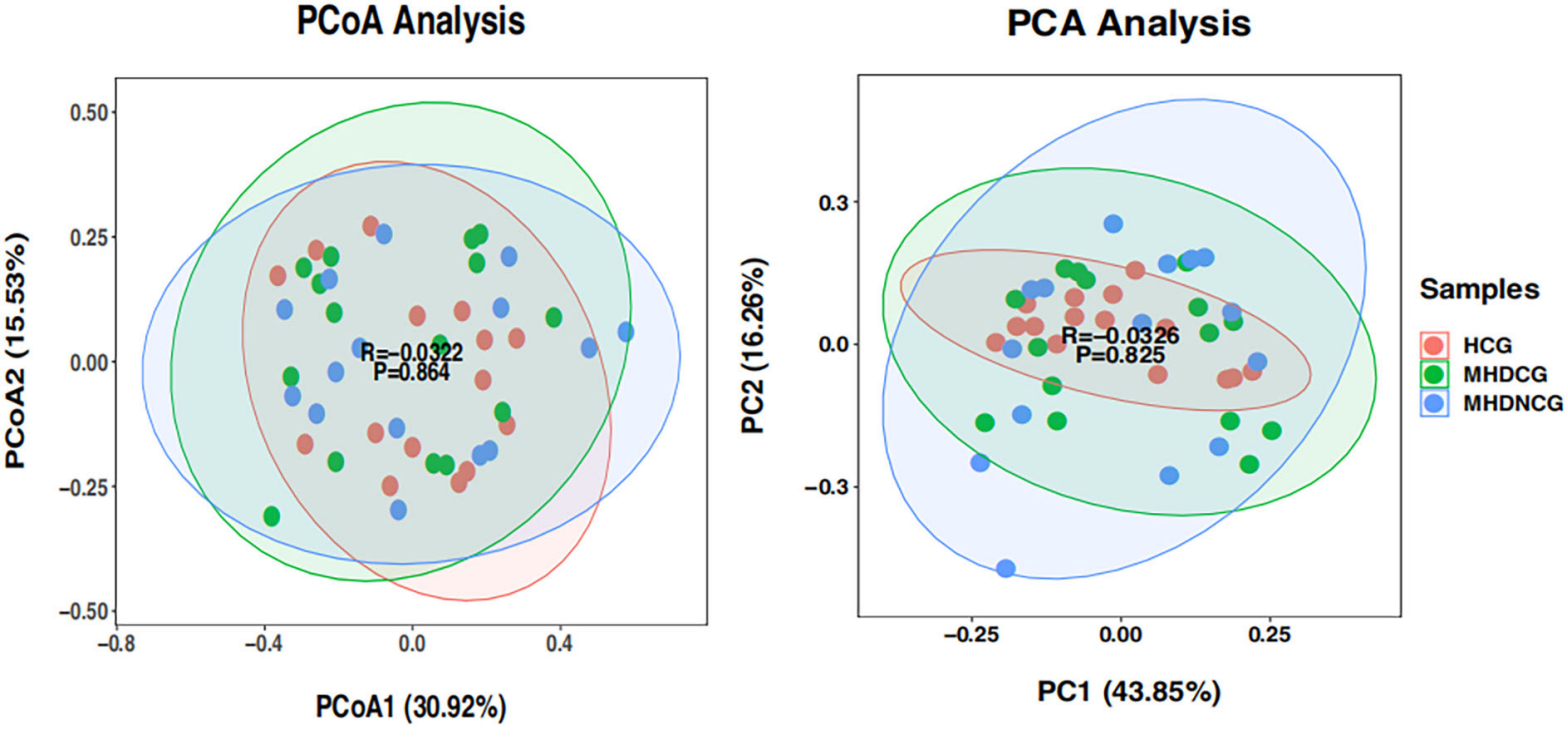
β-diversity analysis. PCoA and PCA analysis revealed no significant difference in the species' variety and abundance between MHDCG, MHDNCG and HCG in Beta diversity analysis (p=0.864, p=0.825).

Both PCoA and PCA analysis revealed no significant difference in the species’ variety and abundance between MHDCG, MHDNCG and HCG in Beta diversity analysis (p=0.864, p=0.825). This suggests that there may be no difference in the variety and abundance of species of the intestinal flora of MHDCG and MHDNCG and HCG (**Figure 3**).

### 3.4 Species difference analysis

#### 3.4.1 Species composition heat map

**Figure 4:**
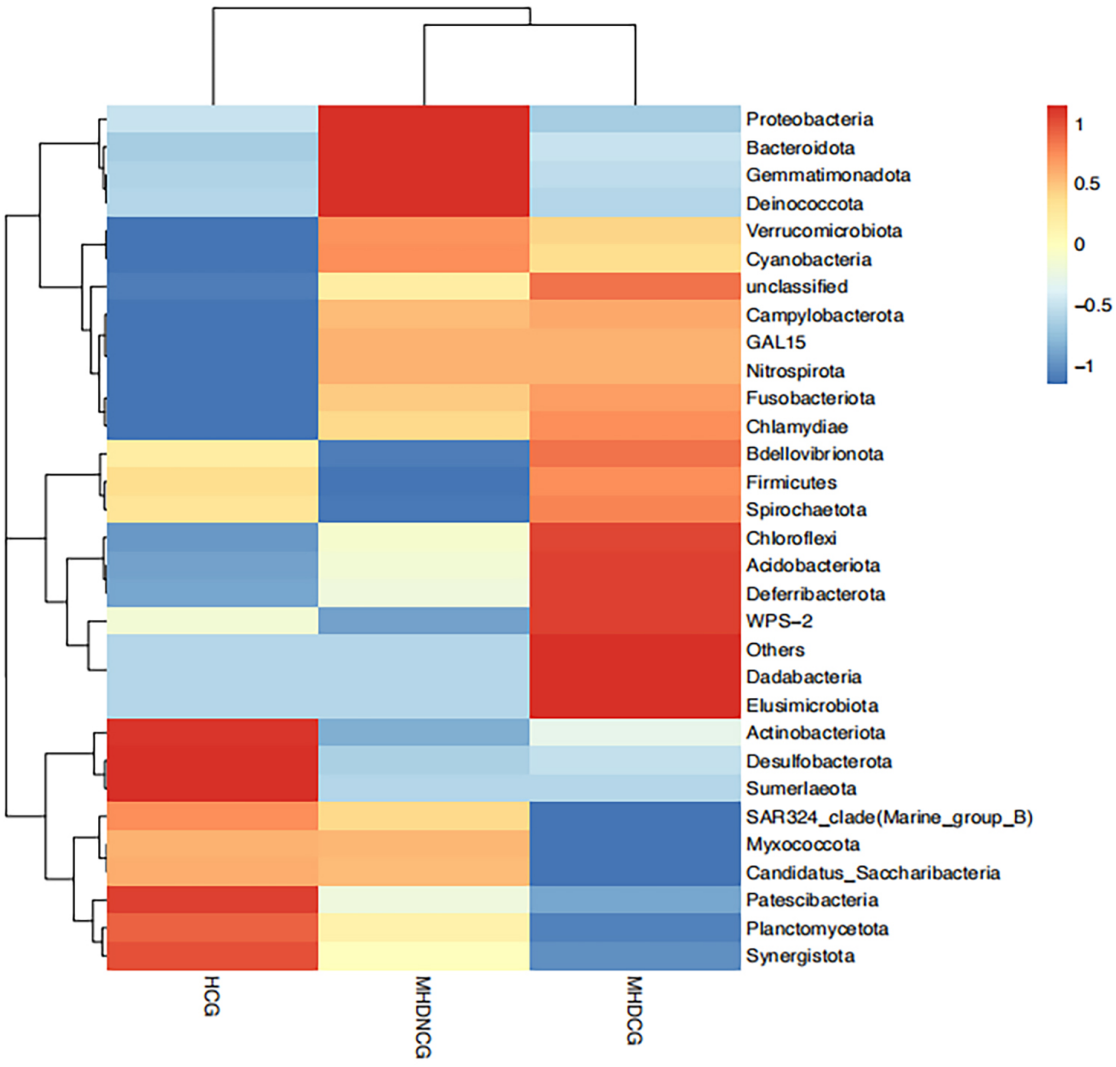
Species composition heat map. Species composition heatmaps revealed less overlap in top30 species abundance between the MHDCG, MHDNCG and HCG groups.

Cluster analyses were used to distinguish between high and low abundance taxonomic units in the community composition of the top30 within each group at the phylum level. Cross-sectional comparisons via species composition heatmaps revealed less overlap in top30 species abundance between the MHDCG, MHDNCG and HCG groups, tentatively suggesting that that the distribution of gut microbes differed among the three groups at the phylum level (**Figure 4**).

#### 3.4.2 Comparison of species abundance

**Figure 5:**
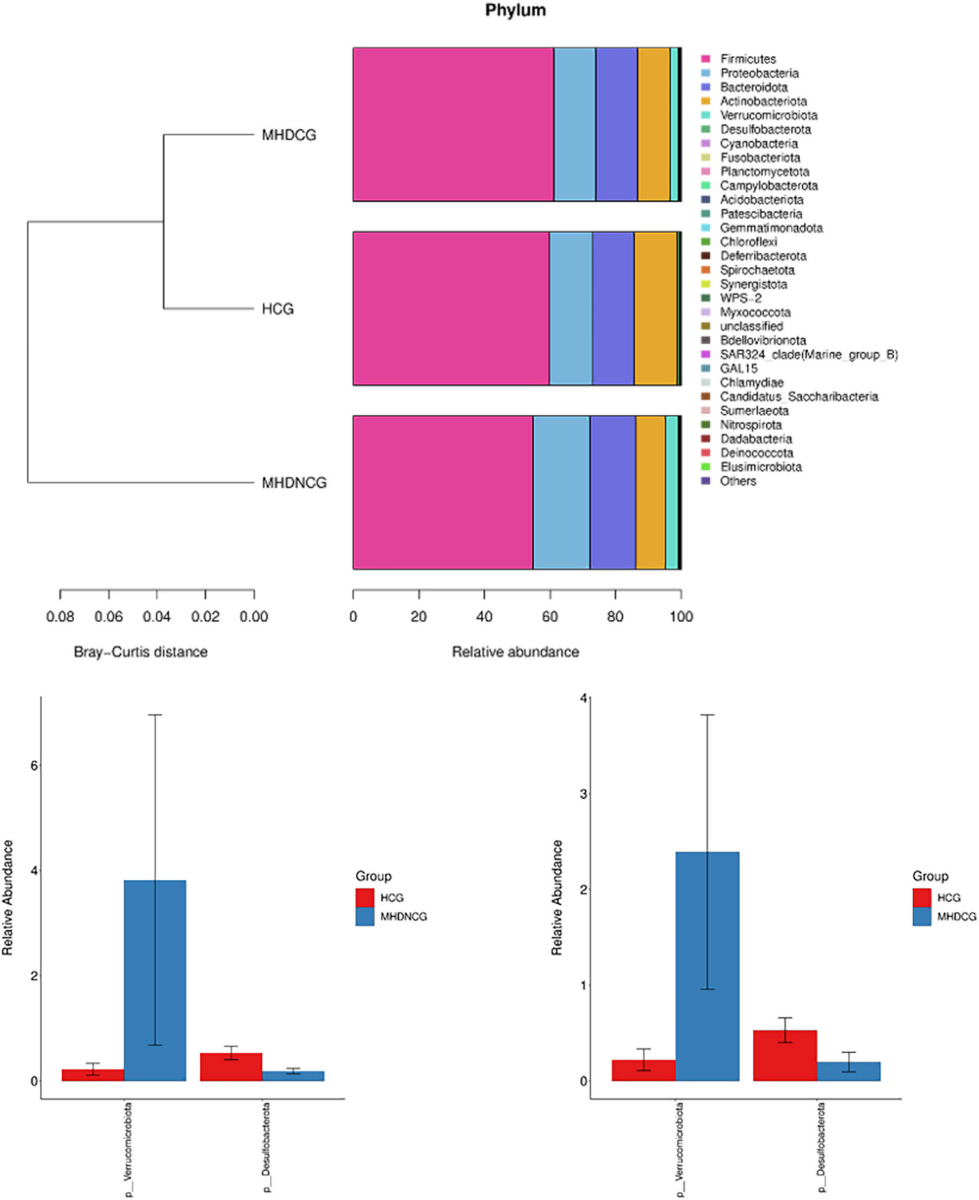
Comparison of species abundance at the phylum level. At the phylum level, the dominant phyla for MHDCG, MHDNCG and HCG were all Firmicutes, Proteobacteria, Bacteroidota, Actinobacteriota and Verrucomicrobiota. The abundance of Verrucomicrobiota in MHD was significantly higher than that in HCG, while the abundance of Desulfobacterota was significantly lower than that in HCG (p<0.05).

At the phylum level, the dominant phyla for MHDCG, MHDNCG and HCG were all Firmicutes, Proteobacteria, Bacteroidota, Actinobacteriota and Verrucomicrobiota. The abundance of Verrucomicrobiota in MHD was significantly higher than that in HCG, while the abundance of Desulfobacterota was significantly lower than that in HCG (p<0.05). There was no significant difference between MHDCG and MHDNCG (**Figure 5**).

**Figure 6:**
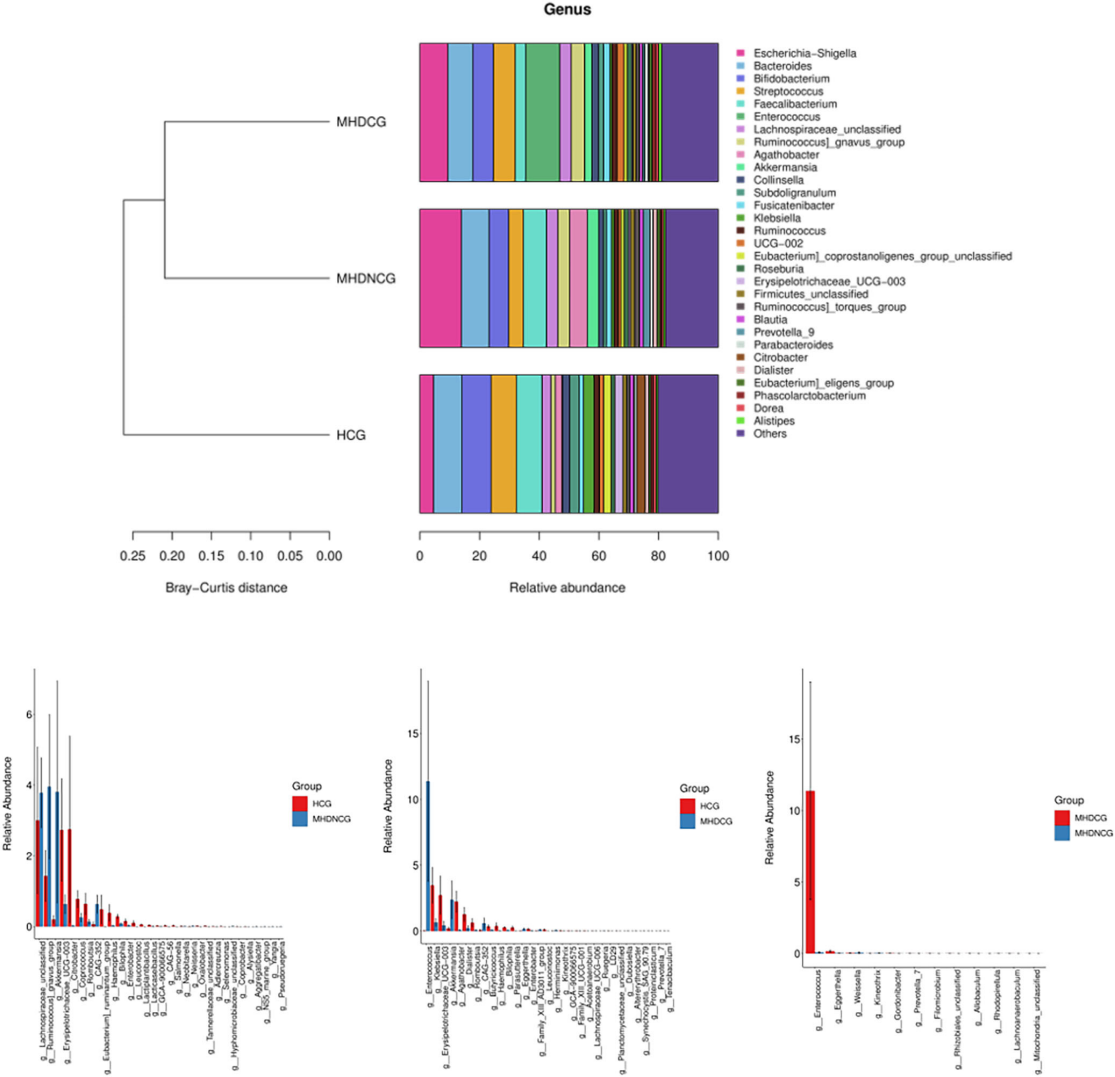
Comparison of species abundance at the genus level. At the genus level, the top 10 dominant genera in the MHDCG group were Enterococcus, Escherichia-Shigella, Bacteroides, Streptococcus, Bifidobacterium, Ruminococcus_gnavus_group, Lachnospiraceae_unclassified, Faecalibacterium, Akkermansia and UCG-002. At the genus level, there were significant differences in the abundance of many species.

At the genus level, the top 10 dominant genera in the MHDCG group were Enterococcus, Escherichia-Shigella, Bacteroides, Streptococcus, Bifidobacterium, Ruminococcus_gnavus_group, Lachnospiraceae_unclassified, Faecalibacterium, Akkermansia and UCG-002. MHDCG showed a significant increase in abundance of Enterococcus, Eggerthella, Ruegeria, Dubosiella, Akkermansia, Filomicrobium, Prevotella_7, Altererythrobacter, CAG-352, Herminiimonas, and significant decrease in abundance of Agathobacter, Haemophilus, Bilophila, Erysipelotrichaceae_UCG-003, Parasutterella, Lachnospiraceae_UCG-006, Leuconostoc, Coprobacter, Dialister, Kineothrix, Butyricicoccus, Romboutsia, Enterobacter and Klebsiella, which compared to HCG(p<0.05). MHDNCG showed a significant increase in abundance of CAG-352, Neisseria, Akkermansia, Lachnospiraceae_unclassified, and a significant decrease in abundance of Bilophila, Haemophilus, Coprococcus, Citrobacter, Lactiplantibacillus, Alysiella, Leuconostoc, Selenomonas, Erysipelotrichaceae_UCG-003, Lacticaseibacillus, Enterobacter, Coprobacter and Neobitarella, which compared to HCG(p<0.05). Compared with MHDNCG, the Enterococcus, Rhizobiales_unclassified, Filomicrobium, Eggerthella, Allobaculum, Prevotella_7, Gordonibacter, Mitochondria_unclassified, Lachnoanaerobaculum were significantly higher and the Kineothrix, Rhodopirellula, Weissella were significantly lower in MHDCG(p<0.05) (**Figure 6**).

#### 3.4.3 LEfSe analysis

**Figure 7:**
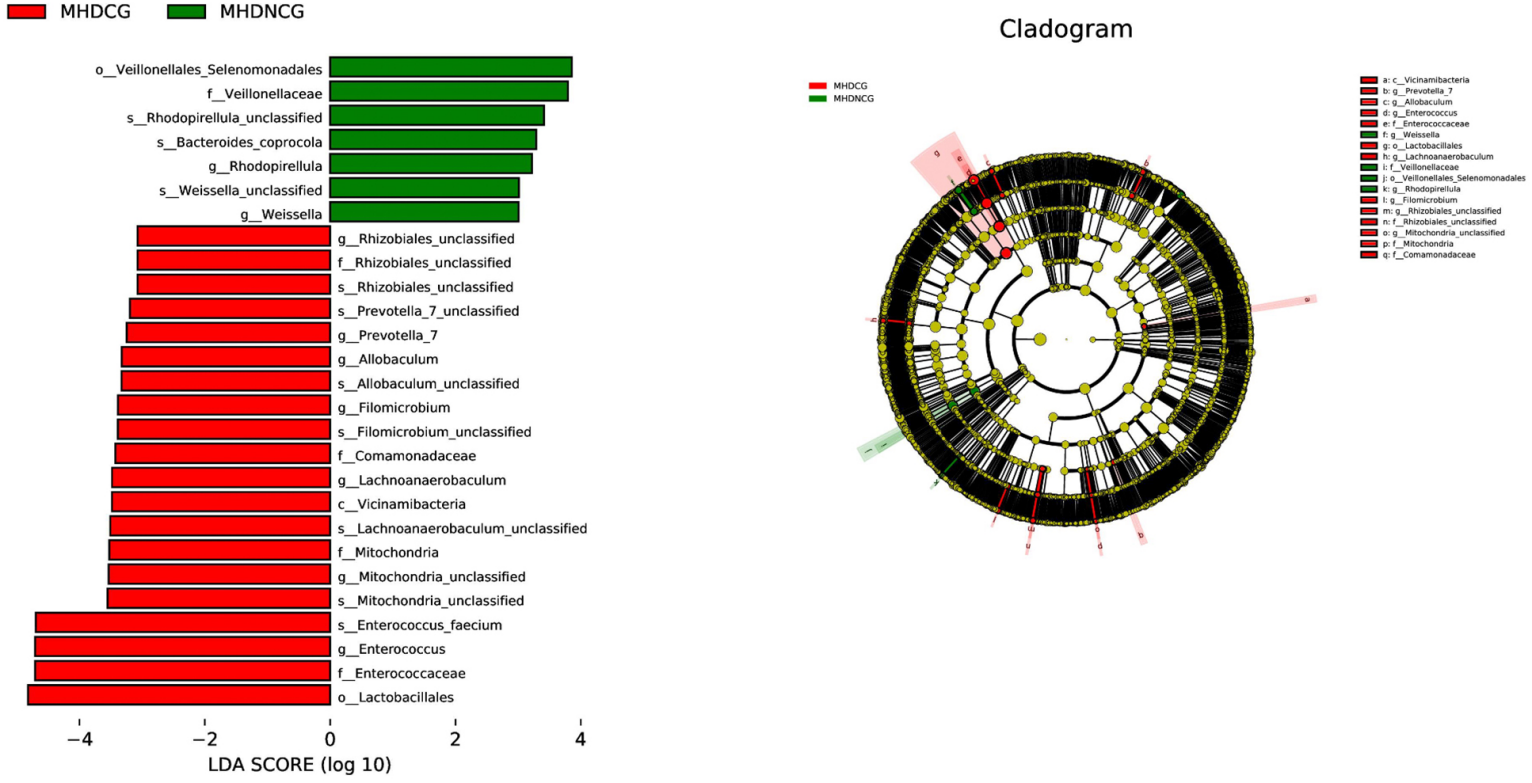
LEfSe analysis. The abundance difference analysis plots for the MHDCG and MHDNCG groups illustrate that 7 gut microbial taxa were significantly more abundant in the MHDNCG group samples, and 20 gut microbial taxa were significantly more abundant in the MHDCG group samples.

The abundance difference analysis plots for the MHDCG and MHDNCG groups illustrate that 7 gut microbial taxa were significantly more abundant in the MHDNCG group samples, and 20 gut microbial taxa were significantly more abundant in the MHDCG group samples. At the genus level, MHDNCG has more abundance in Rhodopirellula and Weissella. MHDCG has more abundance in Rhizobiales, Prevotella_7, Allobaculum, Filomicrobium, Lachnoanaerobaculum, Mitochondria, Enterococcus (**Figure 7**).

### 3.5 Predictive functional analysis

**Figure 8:**
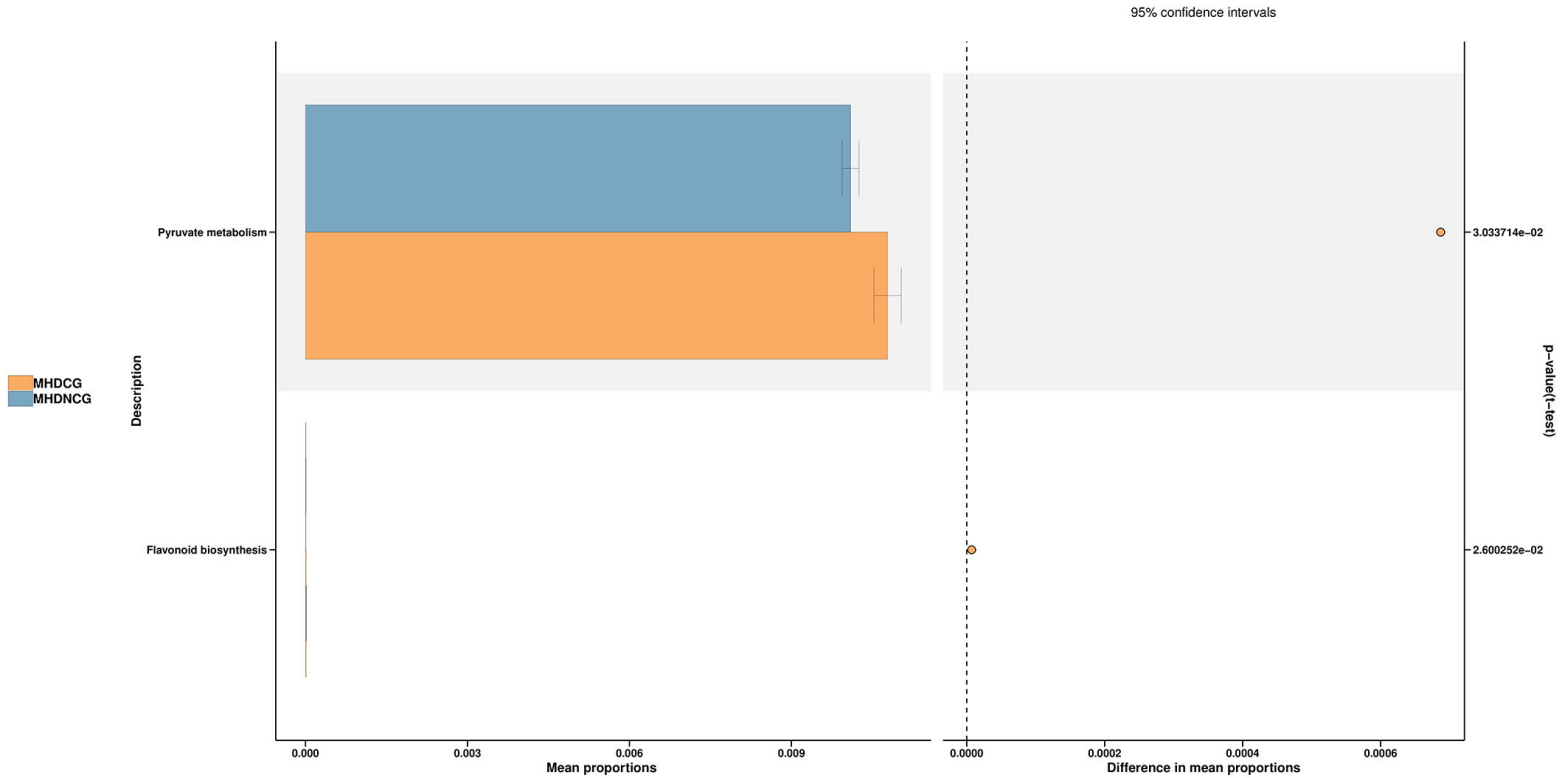
Predictive functional analysis. The PICRUSt2 analysis revealed that compared with MHDNCG, MHDCG was significantly enriched in pathways related to pyruate metabolism, flavonoid biosynthesis.

The species’ functions in the gut microbiota of both the groups were predicted and analyzed based on the amplified sequencing data, using the PICRUSt2 analysis tool. The PICRUSt2 analysis revealed several potential pathway alterations in the microbial communities. Compared with MHDNCG, MHDCG was significantly enriched in pathways related to pyruate metabolism and flavonoid biosynthesis (**Figure 8**).

## 4 Discussion

Maintenance hemodialysis is the most commonly used treatment for uremia, which significantly prolongs the survival of ESRD patients. However, this treatment is inevitably accompanied by a variety of complications that plague patients. Specifically, hemodialysis patients are prone to chronic gastrointestinal diseases such as constipation due to the inability of the kidneys to completely remove toxins, uric acid, oxalic acid and other substances, resulting in the accumulation of toxins in the intestinal tract, accompanied by changes in intestinal function and microbiota. A recent systematic evaluation showed that the most prevalent gastrointestinal symptom in patients receiving dialysis for ESRD was constipation, with prevalence ranging from 1.6% to 71.7% in HD patients (Zuvela et al., 2018).

Gut microorganisms are crucial to the health of the host and the occurrence and development of diseases. The total gut microbial genome is known as the “second human genome”, and tens of thousands of diverse gut microbial communities inhabit the human gut, which are involved in a variety of physiological activities of the host and play an important role in the health of the human body. In recent years,16SrRNA and macro-genomics sequencing technologies have been widely used to the research of human intestinal microbiome, especially to explore the association between intestinal microbiome and cardiovascular, neurological, renal and other diseases. Gastrointestinal disorders are even more closely linked to intestinal flora. The process of digestion and absorption of food in the body and its eventual transformation into feces cannot be achieved without the help of bacteria in the gastrointestinal tract. Therefore, we speculate that the occurrence of constipation in hemodialysis patients may also be related to abnormal intestinal flora. Our trial used 16SrRNA sequencing to analyze the diversity of gut microorganisms in Chinese patients on maintenance hemodialysis with constipation. Macrogenomic studies have shown that Bacteroidota and Firmicutes are the two most dominant bacterial types among human microorganisms, followed by other bacteria such as Proteobacteria, Actinobacteriota, Fusobacteria and Verrucomicrobiota (Adak and Khan, 2019). Our study also demonstrated that Bacteroidota, Firmicutes, Proteobacteria, Actinobacteriota and Verrucomicrobiota are the major phylums in hemodialysis patients and normal healthy individuals. This suggests that to some extent the human gut flora is stable and similar. However, we found that MHDCG had a reduced diversity of gut flora and a heterogeneous distribution of flora compared to the MHDNCG and the HCG, suggesting that the distribution of the gut microbial community is shifted to some extent in patients of MHDCG.

In our study, some potentially pathogenic bacteria (Enterococcus, Eggerthella, Gordonibacter) were more abundant in the constipated group. These bacteria are often responsible for causing inflammation and infection. Studies have shown that Enterococcus exhibit intrinsic resistance to many antimicrobial drugs, such as compound-boosted sulfonamides, cephalosporins, clindamycin, and low-concentration aminoglycosides. Enterococci are regarded as one of the most important hospital infection pathogens among gram-positive bacteria, and their infections are most commonly urinary tract infections (Pan SX, 2008). Gordonibacter is an opportunistic pathogen whose increased abundance is associated with the emergence of enteritis (Pei et al., 2019). The relative abundance of Gordonibacter was significantly and positively correlated with the levels of inflammatory factors such as IL-1, IL-6 and IL-8 (Zhang et al., 2023). Eggerthella can cause bloodstream infections and is considered an opportunistic human pathogen. Eggerthella colonization promotes intestinal Th17 activity and thus induces intestinal inflammation (Alexander et al., 2022). These bacteria raise the chances of inflammation in MHDCG patients. There have been many studies showing a correlation between constipation and inflammation. Mean IL-6, IL-12 and neopterin levels were significantly higher in constipated children than in healthy controls (Cıralı et al., 2018). Constipation correlated with serum IgA antibody titers, while serum IgA levels were positively correlated with ESR values (Strati et al., 2016). In recent years, it has been found that there are structural and functional changes in the intestinal mucosal barrier in patients with chronic constipation, so its role in the pathogenesis of constipation has received widespread attention (Ohkusa et al., 2019). A variety of factors, including bacterial infection (Bischoff et al., 2014), can cause damage to the intestinal mucosal function, increasing intestinal permeability, the intestinal tract loses its normal barrier function, and pathogenic bacteria can take advantage of the situation, resulting in disorders of intestinal flora and abnormal bowel function. Thus, hemodialysis patients with low immunity are more likely to stimulate an inflammatory response once they are attacked by these disease-causing bacteria, leading to intestinal dysfunction and triggering a series of diseases such as constipation.

An animal study found Enterococcus abundance negatively correlates with 5-hydroxytryptophan (5-HT) levels (Hao et al., 2023). Another study found Prevotella to be negatively associated with 5-HT levels (Yang et al., 2023). 5-HT is a prominent neurotransmitter that is highly abundant in the gut and is the driver of the peristaltic reflex (Heredia et al., 2013). And our findings found elevated Enterococcus, Prevotella in MHDCG, which is consistent with the above findings. This suggests that Enterococcus, Prevotella may reduce 5-HT levels, which exacerbates the process of constipation in hemodialysis patients. An RCT of critically ill patients found that exogenous probiotics reduced Enterococcus abundance and effectively relieved constipation (Wang et al., 2021). Similarly, another study found that treatment with herbs reduced Prevotella abundance, activated the 5-HT-cAMP-PKA signaling pathway and improved gastrointestinal motility (Wang et al., 2023). This reminds to us that reducing the abundance of Enterococcus and Prevotella by probiotics, FMT, etc., or activating the 5-HT pathway by 5-HT 4-receptor agonists, may be one of the ways to treat constipation in patients undergoing hemodialysis.

Eggerthella has been associated with autoimmune diseases such as asthma (Wang et al., 2018), multiple sclerosis (Cekanaviciute et al., 2017), inflammatory bowel disease (Alexander et al., 2022)and rheumatoid arthritis (Chen et al., 2016). An animal experiment verifies the role of Eggerthella in the production of serum uremic toxins and in aggravating the development of renal disease (Wang et al., 2020). Another study (The effect of constipation on urinary toxin levels in patients with chronic kidney disease and the evidence of Chinese medicine, 2023) showed that constipation can exacerbate urinary toxin accumulation in CKD patients, and the more severe the constipation, the greater the urinary toxin accumulation. The gut and kidneys are closely related pathophysiologically. Increased intestinal permeability allows a variety of pathogens to enter the bloodstream, leading to aberrant immune activation and facilitating the progression of renal disease, while deterioration of renal function leads to the accumulation of toxins in the body, causing disturbances in the internal environment and exacerbating the disruption of the intestinal barrier. Combining the above studies with ours, we hypothesize that Eggerthella may cause constipation and exacerbate the accumulation of urinary toxins.

The gut microbiota regulates peristalsis by the release of microbial metabolites or fermentation end products. The three main groups of bacterial metabolites include Bas, short-chain fatty acids (SCFAs) and tryptophan metabolites. SCFAs are major metabolites produced by specific colonic anaerobes after fermentation of dietary fiber and resistant starch. Short-chain fatty acids stimulate the production of glucagon-like peptide-1 and peptide YY, modulate the release of 5-HT, and increase gastrointestinal motility. SCFAs mainly consist of acetic acid, propionic acid, and butyrate, which also directly modulates the enteric nervous system and controls gastrointestinal motility (Soret et al., 2010). It has been reported that butyrate is mainly produced by Lachnospiraceae, Ruminococcaceae, Faecalibacterium, prausnitzii, Prevotella, Roseburia, and Clostridium (Pryde et al., 2002). However, our study found no significant difference in the abundance of these bacteria between the two groups, except that Prevotella was elevated in MHDCG. In addition, there was an increase in Allobaculum and Lachnoanaerobaculum in MHDCG, which are also capable of producing butyrate. It seems contrary that butyrate promotes intestinal peristalsis, whereas butyrate-producing bacteria are instead increased in MHDCG. This may be due to the differences in race, geography, and underlying disease between our experimental population and the participants in the above studies. However, there are also studies that showed non-physiological high levels of short-chain fatty acids in the gut may lead to gastrointestinal symptoms, including a constipated state, by altering the secretion of mucin secretions from cuprocytes (Barcelo et al., 2000) and inhibiting intestinal smooth muscle contraction (Squires et al., 1992) mediated by the release of peptide YY from enteroendocrine cells (Cherbut et al., 1998). Based on the above studies, we hypothesized two possible mechanisms for the elevation of short-chain fatty acid-producing bacteria (Prevotella, Allobaculum, Lachnoanaerobaculum) in MHDCG. First, constipation leads to an increase in retained dietary fiber and resistant starch in the colon, causing compensatory elevation of short-chain fatty acid-producing bacteria. Second, the abnormal elevation of short-chain fatty acids leads to constipation. Colonic epithelial cell mitochondria can catalyze butyrate to nicotinamide adenine dinucleotide (NADH) to participate in the oxidative phosphorylation (OXPHOS) process. OXPHOS produces high oxygen consumption and maintains a hypoxic environment in the intestinal lumen, which helps to maintain a predominantly anaerobic gut microbial community (Litvak et al., 2018). In our study, both mitochondria and butyrate-producing bacteria were elevated in MHDCG, which seems to be a process of interaction. In addition to this, an important function of mitochondria is to supply energy to the cell, and the increase in mitochondria may be an adaptive response caused by the increased functional load of the gut (Qiao et al., 2020; Choi et al., 2023).

Our study found that pyruvate metabolism was stronger in MHDCG than in MHDNCG. An animal experiment shows significantly higher levels of pyruvate metabolism in the intestinal flora of a rat model of constipation (Study on the effects of fluid-enhancing soup on intestinal flora and host metabolism in rats with fluid deficiency and senile constipation, 2018), and a serum metabolic profiling study also showed significant enrichment of the pyruvate metabolic pathway in constipated patients (Xu et al., 2022). These findings are consistent with our results. The pyruvate metabolism pathway belongs to energy metabolism, and the high expression of this pathway suggests that constipation may lead to accelerated carbohydrate metabolism and increased energy expenditure. We also found a significant enrichment of flavonoid biosynthesis pathways in MHDCG compared to MHDNCG. Recent studies have shown that flavonoids and their derivatives can be antioxidants, reduce low-density lipoprotein, inhibit thrombosis, inhibit tumors, protect liver cells, anti-fatigue and other effects (Physiological functions of soybean isoflavones and their application prospects, 2002; Yaru et al., 2022). Flavonifractor uses gamma-aminobutyric acid (GABA) as a growth substrate, and hence reduces the amount of GABA in the gut (Strandwitz et al., 2019). GABA has a variety of regulatory roles in the intestinal tract, including promoting peristalsis and gastric emptying (Hyland and Cryan, 2010). Thus, enrichment of flavonoid biosynthesis pathways may cause constipation. However, there are study showing that flavonoids in *Amomi Fructus* improved constipation symptoms in mice by modulating the gut microbiota and related metabolites (Hu et al., 2023), contrary to our findings. It is possible that this is an effect of the differences between the human and mice species. There is a lack of reports on the association between flavonoid biosynthesis pathways and constipation, and the association needs to be further investigated.

There are potential limitations to this study. First, although this study characterized the microbial composition of MHDCG patients, the population included in this study was limited, all from Hangzhou, China. Ethnic and geographic limitations may have biased the results somewhat. Second, due to the strict conditions of sample collection and preservation, we only collected 15 samples from each group. Third, although our statistical results showed no statistical difference between MHDCG and MHDNCG in the use of medications affecting intestinal motility, patients’ use of lactulose and polyethylene glycol-4000 all have the potential to affect intestinal microbes (Bouhnik et al., 2004). In our study, the interference of these drugs was not completely avoided. In addition, we lacked metabolomics studies, and the mechanism between differential flora and maintenance hemodialysis constipation was not adequately investigated. We look forward to more multi-center, large-sample studies on the intestinal flora of patients with maintenance hemodialysis constipation in the future.

## 5 Conclusions

This study describes for the first time the gut microbiome characteristics of patients with maintenance hemodialysis constipation. The results showed that MHDCG differed significantly from MHDNCG and HCG in the abundance of some intestinal bacteria, which may be the direction for future FMT treatment. By studying these differential bacteria, we believe that constipation in hemodialysis patients may be associated with a variety of mechanisms, including inflammatory responses induced by potentially pathogenic bacteria, decreases in 5-HT, interactions between urinary toxins and intestinal function, and abnormal increases in short-chain fatty acids. However, there are limitations in this study and the understanding of the mechanisms of constipation in hemodialysis patients lacks direct evidence, which needs to be further explored by more multi-center and large-sample studies in the future.

## Data Availability

The datasets presented in this study can be found in online repositories. The names of the repository/repositories and accession number(s) can be found below: https://www.ncbi.nlm.nih.gov/, PRJNA1159710.
