# Supplementary material for: Intestinal microbiome changes and mechanisms of maintenance hemodialysis patients with constipation
Source: Front Cell Infect Microbiol. 2024 Nov 11;14:1495364. doi: 10.3389/fcimb.2024.1495364 (PMC11586350; doi:10.3389/fcimb.2024.1495364)
Supplement: Supplementary file 1 [file DataSheet1.pdf]

罗马 IV 便秘诊断问卷调查表

1. 最近 3 个月，在未使用通便药或灌肠剂的情况下，你每周排便次数为多少？a. ≥1 次/天=0
b. ≥3 次/周=1
c. ≤3 次/周=2
2. 最近 3 个月，你排干硬便或干球便有多频繁？
a. 从不=0
b. 有问题（≤50%）=1
c. 严重问题（≥50%）=2
3. 最近 3 个月，你是否感到排便费力？
a. 从不=0
b. 有问题（≤50%）=1
c. 严重问题（≥50%）=2
4. 最近 3 个月，你是否感到排便后仍有排便不尽感？
a. 从不=0
b. 有问题（≤50%）=1
c. 严重问题（≥50%）=2
5.最近 3 个月，你是否排便时感到大便在肛门处有梗阻/堵塞感？
a. 从不=0
b. 有问题（≤50%）=1
c. 严重问题（≥50%）=2
6. 最近 3 个月，排便时你是否需要手法辅助（如用手指或者借助开塞露等）？a. 从不=0
b. 有问题（≤50%）=1
c. 严重问题（≥50%）=2
7.你是否曾被诊断为“肠易激综合征”？
a. 是
b. 否
8.不使用通便药物情况下，你是否会出现稀便？
a. 是
b. 否
9.对于便秘情况（问题 1～8），你是否已经持续 6 个月及以上？
a. 是
b. 否

符合问题 9 和 1～6 中两条及两条以上，排除问题 7 和 8 即可诊断为便秘。

便秘程度量化评估结果：总分

Rome IV Constipation Diagnostic Questionnaire

1. In the last 3 months, without the use of laxatives or enemas, how many times per week did you have a bowel movement? a. ⩾ 1 time/day = 0
b. ⩾3 times/week = 1
c. ⩽3 times/week = 2
2. In the last 3 months, how often did you pass dry, hard or dry bulbous stools?
a. Never = 0
b. Problematic (⩽50%) = 1
c. Serious problem (⩾50%) = 2
3. In the last 3 months, did you feel strained to pass stools?
a. Never = 0
b. Problematic (⩽50%)=1
c. Serious problem (⩾50%)=2
4. In the last 3 months, did you feel a sense of incomplete bowel movement after defecation?
a. Never = 0
b. Problematic (⩽50%)=1
c. Serious problem (⩾50%)=2
5. In the last 3 months, did you feel a sense of obstruction/blockage of stool at the anus during defecation?
a. Never = 0
b. Problematic (⩽50%)=1
c. Serious problem (⩾50%) = 2
6. In the last 3 months, did you need manual assistance (e.g., with fingers or with the help of a corkscrew, etc.) to have a bowel movement? a. Never = 0
b. Problematic (⩽50%) = 1
c. Serious problem (⩾50%) = 2
7. Have you ever been diagnosed with "irritable bowel syndrome"?
a. Yes
b. No
8. Do you have loose stools without laxatives?
a. Yes
b. No
9. Has constipation (Q1-8) lasted for 6 months or more?
a. Yes
b. No

The diagnosis of constipation can be made if two or more of the questions 9 and 1 to 6 are met and questions 7 and 8 are excluded.
Quantitative assessment of the degree of constipation: Total score
